# Single-step phase identification and phase locking for coherent beam combination using deep learning

**DOI:** 10.1038/s41598-024-58251-z

**Published:** 2024-03-29

**Authors:** Yunhui Xie, Fedor Chernikov, Ben Mills, Yuchen Liu, Matthew Praeger, James A. Grant-Jacob, Michalis N. Zervas

**Affiliations:** Optoelectronics Research Centre, University of Southampton, Southampton, UK

**Keywords:** Fibre lasers, Fibre optics and optical communications, Liquid crystals

## Abstract

Coherent beam combination offers a solution to the challenges associated with the power handling capacity of individual fibres, however, the combined intensity profile strongly depends on the relative phase of each fibre. Optimal combination necessitates precise control over the phase of each fibre channel, however, determining the required phase compensations is challenging because phase information is typically not available. Additionally, the presence of continuously varying phase noise in fibre laser systems means that a single-step and high-speed correction process is required. In this work, we use a spatial light modulator to demonstrate coherent combination in a seven-beam system. Deep learning is used to identify the relative phase offsets for each beam directly from the combined intensity pattern, allowing real-time correction. Furthermore, we demonstrate that the deep learning agent can calculate the phase corrections needed to achieve user-specified target intensity profiles thus simultaneously achieving both beam combination and beam shaping.

## Introduction

In recent decades, high-power fibre lasers have found many new applications due to significant advancements in their output power^1–4^, however, surpassing kilowatt average power levels poses challenges due to the inherent physics of optical fibre transmission. Nonlinear effects such as stimulated Raman scattering, stimulated Brillouin scattering, and the optical Kerr effect rapidly become prominent as the power is scaled; whilst increasing the mode area can mitigate some issues, it introduces new challenges such as transverse mode instability^5^. Coherent beam combination (CBC)^6–9^, where light from multiple fibres is combined, has therefore emerged as a technique for enhancing total average power without increasing the power handling requirement for individual fibres. Since the resultant spatial intensity profile from a CBC system is highly dependent on the relative phases of the combined fibres, CBC can also offer a means of beam shaping, e.g. for creation of exotic beam profiles^10^ and for non-mechanical focal plane adjustment^11^. The ability to identify and compensate the fibre’s (time varying) relative phases is critical in the production of consistent and high-power laser output, and so development of appropriate phase-locking control systems has become an important research topic for power scaling and optimisation of laser output in CBC systems.

Whilst methods exist for identification of the fibre phases through spatial interference or temporal beating^12,13^, a simpler approach is desired. Direct interrogation of the combined intensity pattern (i.e. with a camera) cannot provide information regarding the phase of each fibre, as only the modulus-squared of the electric field is recorded. Whilst iterative techniques such as Gerchberg–Saxton, hybrid input–output (HIO)^14^, hill-climbing^15,16^, stochastic gradient descent^17^ and reinforcement learning^18–22^ can all be used to identify the phase values, a single step phase correction process is required due to the continuously changing phase values for each fibre.

Deep learning^23,24^ offers the potential for identifying solutions to complex problems directly from experimental data, and has therefore been applied widely across the field of laser development^25^ and laser materials processing^26,27^. Understandably, deep learning has also been applied to CBC. Recent results include experimental demonstrations for the control of 107 beams^28^ and a 7-channel 7 kW combination^29^ using stochastic gradient descent, and momentum based gradient optimisation^30^. Such approaches rely on the maximisation of power transmitted through a focal-plane aperture, and therefore offer an elegant approach for maximising power-in-the-bucket for a given CBC system. Whilst stochastic gradient descent is an iterative approach, the simplicity allows for rapid iteration speed (typically > 1 MHz), but the capability of this approach generally decreases rapidly as the number of fibres is increased^16^ and hence is not considered suitable for larger numbers of fibres. Gradient based optimisation also generally requires the introduction of random noise fluctuations to assist in the optimisation process. However, whilst such approaches allow for maximisation of the power in a single interference peak, many applications require specific spatial intensity profiles. To enable beam shaping in a CBC configuration, it is necessary to identify the phase of each fibre, in a single step, so that phase corrections can be applied and the desired beam shape be created. Therefore, there has been considerable interest in techniques that enable the identification of the fibre phases directly from the combined intensity pattern that is recorded on a camera.

However, recovering the phase profile from a spatial intensity profile leads to the fundamental challenge where there may be many (or even infinite) phase profiles that can result in a specific spatial intensity profile^31,32^. Presented solutions include interference with a reference beam^33^, use of a diffractive optical element^34^, observation of the combined beam away from the focal plane to achieve a single interference peak^35^, and two-step phase identification and optimisation^36^.

In 2022 we presented a theoretical solution for unlocking single-step bespoke beam shaping in a CBC system through the application of deep learning to the combined intensity patterns^37^ (with analysis of the robustness of the approach in^38^). We also showed how two neural networks could be used in tandem, to solve an important inverse challenge associated with CBC, namely whether a desired intensity pattern is physically possible on a given experimental setup. In this work, we present an experimental validation of these theoretical results and demonstrate bespoke beam shaping in the presence of random phase noise by using seven beams created by a spatial light modulator. These results offer another step towards implementation of real-time beam shaping on high-power fibre laser systems.

## Experimental setup

A spatial light modulator (SLM) (Thorlabs, EXULUS-HD1/M, 1920 × 1080) was used in conjunction with a stabilised HeNe laser (Thorlabs, HRS015B, 632.992 nm, 1.2 mW, linear polarisation). The incident beam was expanded to approximately twice the size of the SLM to ensure that the SLM was illuminated with relatively uniform intensity. Seven circular regions were defined on the SLM in order to simulate a tiled aperture coherent beam combination configuration with seven fibres in a hexagonal close packed arrangement. The circular regions had radii of 199 pixels and adjacent circles had a centre-to-centre distances of 400 pixels (pixel pitch is 6.5µm). Individual phase offset values between −π and + π (relative to the centre circle) could be applied to each of the outer circles. The phase pattern corresponding to a horizontal blazed grating was additionally superposed on the circular regions whilst a vertical blazed grating phase modulation was applied to all pixels outside of the circles. This ensured angular separation between light from the circular regions and background light (either reflected from the physical boundary of the SLM or from pixels outside of the circular regions).

**Figure 1:**
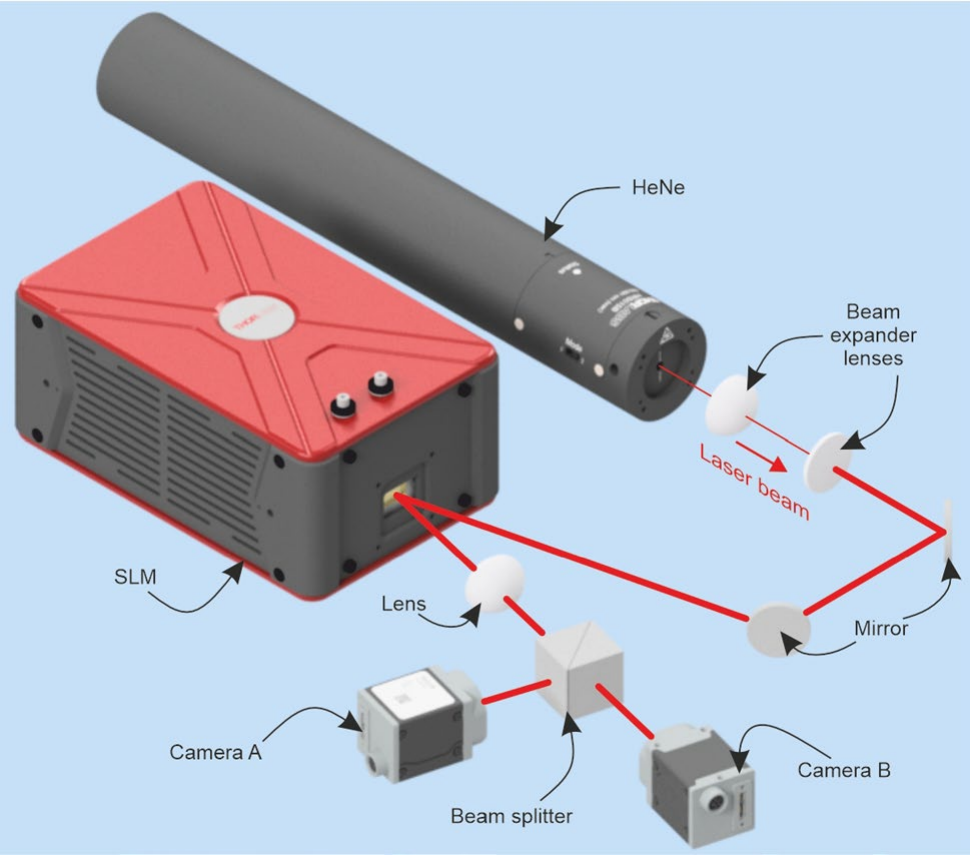
The output of the HeNe laser was expanded and directed onto the SLM to create seven parallel beams with controllable phase values. These beams were brought to a focus with a lens, and the intensity profile was measured by camera A (before the focal plane) and camera B (at the focal plane).

Light reflected from the SLM was brought to a focus using a lens (f = 30cm) this caused light from the circular regions to overlap spatially, simulating the CBC process. The resulting spatial intensity profile was measured at two positions simultaneously, using a beam splitter and two cameras (Basler, a2A4505-18uc, resolution 4512 × 4512, pixel size 2.74 × 2.74 µm, RGB, used as 8bit). The experimental setup is presented in Fig. 1, which shows the two cameras are referred to as "camera A" and "camera B", where camera A was positioned 5cm from the focal plane and camera B was positioned at the focal plane.

## Neural network training and validation

Training data were experimentally collected by randomly changing the phase of each of the outer six circles on the SLM, whilst simultaneously recording images from camera A and camera B. The phase delay applied at the central circle was kept fixed (defined as 0π) whilst the relative phase offsets of the outer six circles were individually sampled from a uniform distribution in the range -π to + π. The integration times for camera A and camera B were 30 ms and 0.1 ms respectively, and were chosen to produce camera images with a high signal-to-noise ratio, but without any image saturation. Data collection for a single training pair consisted of the creation of a set of random phase values followed by recording of the images on the two cameras. The camera images were cropped to 1024 × 1024 pixels, down-sampled to 256 × 256 using bilinear interpolation, and saved as a single-channel 8-bit PNG image. Due to the 4 × down-sampling each pixel in the processed dataset, and in the camera images presented below, corresponds to a region with physical size 10.96 × 10.96 µm at the camera sensor.

A critical component in the presented beam shaping process is a neural network that can identify the individual fibre phases from their combined intensity pattern. Here, two separate neural networks were trained to compare the effectiveness of phase identification away from the focal plane (camera A) and at the focal plane (camera B). Both neural networks had the same architecture and hyperparameters, with the only difference being the training materials (i.e., images from camera A or from camera B). The neural network architecture was based on MobileNetV3-Small^39^ with a width multiplier of 1.0, and the following three key changes: firstly, the input image size was changed to 256 × 256 pixels, from 224 × 224 pixels. Secondly, the dimension of the output layer was changed from N × 1000 to N × 6, to enable the simultaneous prediction of six phase values. Thirdly, the loss function was changed to the trigonometric function 2–2 × cos(θ − φ), where θ and φ were the predicted and experimental phase values respectively. A cyclical loss function was adopted to address the ambiguity in predicting continuous values with a periodic nature, as shown in previous work^40^. The Adam optimizer^41^ was used with a learning rate ${\alpha }_{t}$ of 0.0001, exponential decay hyper-parameters ${\beta }_{1}$ of 0.900 and ${\beta }_{2}$ of 0.999 and no weight decay. A 20% dropout rate^42^ was applied to improve the generalization. The MobileNet architecture was chosen for this work as it was designed with computational efficiency and latency in mind. The inference time for the prediction of six phase values from an experimental camera image was 3.8 ± 0.5 ms, as measured on the workstation that controlled the experiment (Microsoft Windows 10, Intel Xeon w5-3423, NVIDIA RTX A4000).

**Figure 2:**
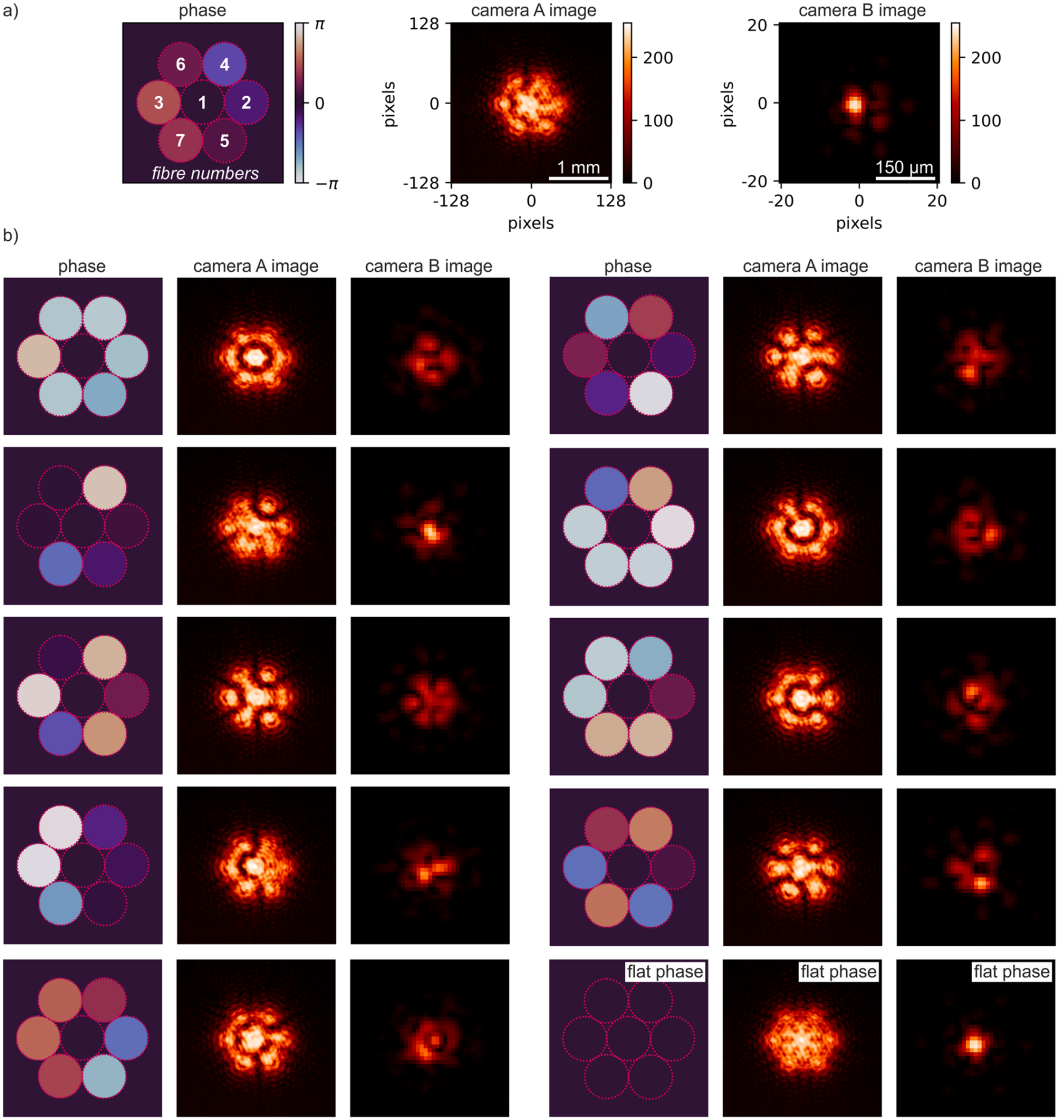
(**a**) Schematic depicting the indices of all simulated fibres and the colour mapping for the phase values used in this work, and the associated experimental images recorded on camera A and camera B. (**b**) Examples of randomly chosen phase values and their corresponding camera A and camera B images, with the final example highlighting the case where all phases are zero; the deviation from perfect sixfold rotational symmetry and the observed spatial intensity distortion in the camera observations can be attributed to the 6° deviation from right-angle between the incidence beam and the SLM.

Camera B data collection took 22 h 14 min, for a total of 26,071 images, with a 90/10% split for training and validation, taking 5 h 26 min to train. Camera A data collection took 17 h 57 min, for a total of 18,114 images, with a 90/10% split for training and validation, taking 6 h 8 min to train. Each neural network underwent 500 epochs of training, encompassing the entire dataset in each iteration. The weights obtained from the final training epoch were saved and subsequently employed in the ensuing experimental procedures (the training curves can be found in the supplementary material in Fig. S1). Although the camera A and camera B images used for neural network training were identical in size and type, there was a noticeable difference in training times. This was a result of variations in data transfer and data synchronisation speeds between the experimental setup and the cloud-based hardware performing the training. Training was completed on a Microsoft Windows 10 computer workstation with an AMD Ryzen Threadripper PRO 3995WX CPU and four NVIDIA RTX A5000 graphic cards. Examples from the experimental data are shown in Fig. 2, where (a) shows a single example with additional labelling and (b) shows ten further examples. As shown in Fig. 2a each item in the dataset has three components: the leftmost image shows a false-colour representation of the relative phase applied to each circular region (see colour bar), dashed lines outline the circular regions and the numbers indicate the fibre indices. (In fact, this data can also be represented as a vector of six floating point numbers). The central and rightmost plots in each set show the cropped and down-sampled images from camera A and B respectively. In these plots the colour bar indicates the number of counts per pixel per exposure time expressed as an 8bit unsigned integer [0–255]. Due to the smaller physical size of the beam at the focal plane, the images for camera B are further cropped to show only the central 40 × 40 pixel region as indicated by the scalebars and axis labels. The images from camera A contain many detailed interference fringe type features, whilst those from camera B contain highly variable lobed patterns. Within Fig. 2b the last example (bottom right) shows the camera images observed for the “flat phase” case, which results in a highly symmetric pattern and the tightest concentration of intensity.

**Figure 3:**
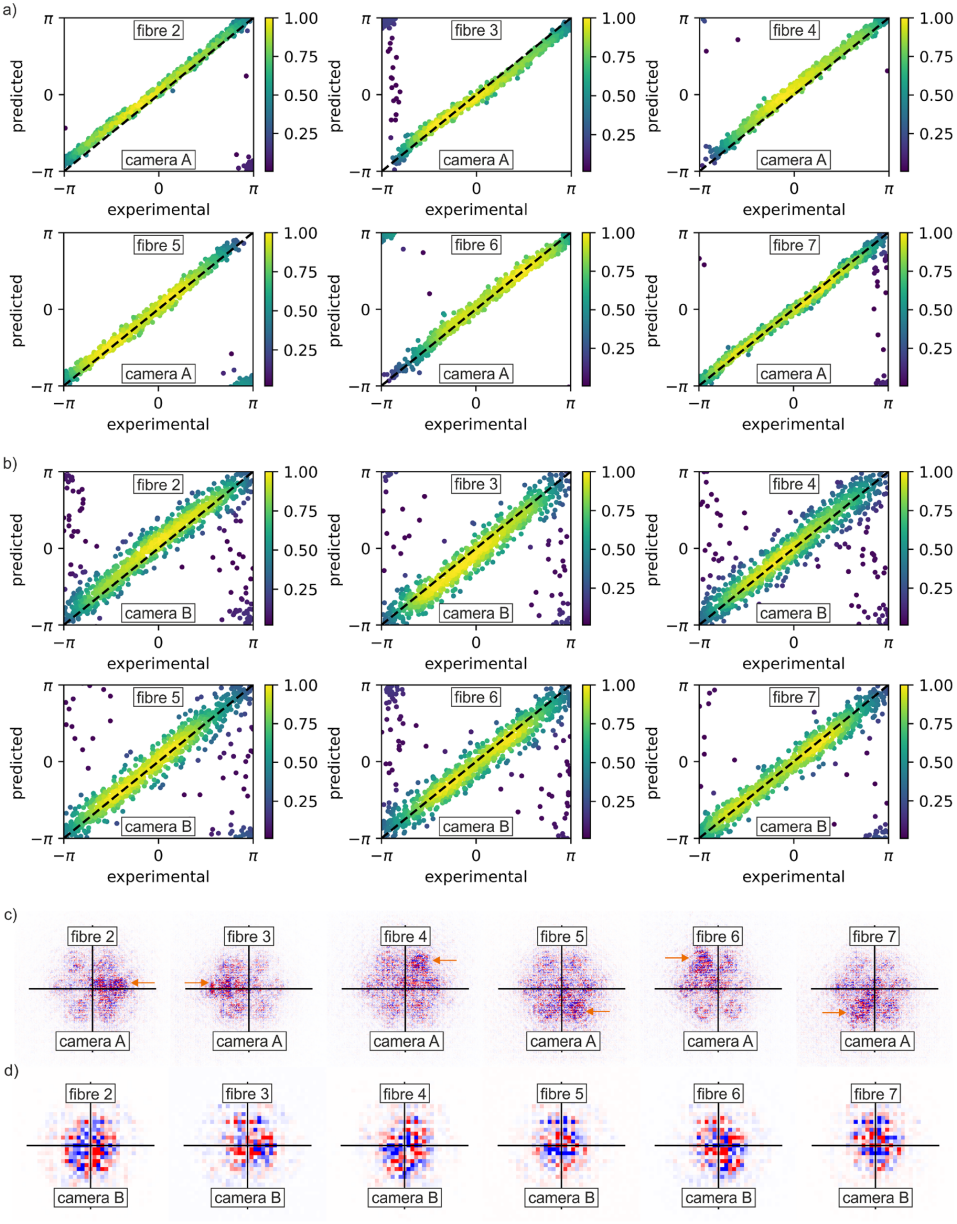
Prediction accuracy for (**a**) camera A (µ = 0.26 ± 0.08 radians) and (**b**) camera B (µ = 0.41 ± 0.21 radians), and relative importance of camera pixels in making phase predictions calculated using gradient analysis for (**c**) camera A and (**d**) camera B. Orange arrows highlight the asymmetry in (**c**).

After training, each of the two neural networks can be used to predict the relative phase offsets of the circular regions, directly from (previously unseen) observations from their respective cameras. Figure 3a analyses the accuracy of predicted phase values, for each simulated fibre, based on the images from camera A; Fig. 3b shows a similar analysis but based on the network and input data associated with camera B. The graphs each plot 1000 datapoints from a real-time test where 1000 images were recorded from each camera, in an experiment conducted the day after the networks were trained. In each case, the x-axis shows the experimental (real) value, and the y-axis shows the prediction by the neural network, where the function y = x is added for visual clarity. The predictions from camera A were found to be considerably more accurate than the ones from camera B, with average prediction errors of 0.26 ± 0.08 and 0.41 ± 0.21 radians, respectively. An important consequence of a smaller region of interest for camera B, is that the associated network was more sensitive to movement in the beam. As such, any undesired and unintended camera misalignment (e.g. camera mechanical drift) that might be experience on a real fibre laser CBC system would affect the network trained on camera B images more severely. Based on testing on images with artificial lateral offsets, the mean phase prediction error increases from 0.26 ± 0.08 radians to 0.32 ± 0.12 radians for the camera A model, and from 0.41 ± 0.21 radians to 0.69 ± 0.37 radians for the camera B model, when the beam is deliberately displaced by half of its full width at half maximum at focus. However, previous work has shown that data augmentation, where artificial lateral shifts are added to the training data^40^, could compensate this effect to some extent. In addition, magnification of the image, to ensure full coverage on the camera, would also likely help.

Figure 3c, d present analysis of the gradients^43^ within the two neural networks which results in "attribution maps" that quantify the relative importance of each pixel to the neural network when it makes its phase prediction. Since each neural network simultaneously predicts the relative phases of six simulated fibres, when provided with a single experimental camera image, six attribution maps can be generated. Figure 3c presents averaged attribution maps for predictions from the camera A neural network and shows clear asymmetry in the gradients (highlighted with orange arrows). The pixels with the greatest importance are consistently found near to the simulated fibre whose phase is being predicted. The asymmetry provides evidence that the neural network has learned which regions of the images contain the most information associated with the phase of each simulated fibre. No such asymmetry was observed in the gradient analysis for the network associated with camera B, positioned at the focal plane, as shown in Fig. 3d. This observation can be explained by Fourier theory, as the spatial information corresponding to the phase values is equally distributed over the spatial extent of the combined beam at the focal plane (camera B), but this is not true at positions away from the focal plane (camera A). This result, demonstrating greater spatial localisation of information within the camera A dataset, perhaps provides a partial explanation for the higher prediction accuracy observed for camera A images. The training data corresponding to camera A and camera B differ in the absolute number of images (18,114 for camera A and 26,071 for camera B), in the appearance of the intensity pattern (camera A had a larger region of interest than camera B), and in the integration time (30 ms for camera A and 0.1 ms for camera B). Whilst a precise comparison between the neural networks trained to identify phase at the two camera positions was not the intention of this work, some broad conclusions can be drawn. Firstly, the differences in integration time can be mostly ignored, as these were chosen to achieve an average maximum intensity that was just below the saturation level of the cameras. Secondly, the performance gap observed between the two neural networks trained with datasets sourced from cameras A and B is unlikely attributable solely to the difference in dataset size. This assertion is supported by the finding that despite the camera B dataset containing more images than the camera A dataset, it still yields a comparatively inferior model in terms of performance. Lastly, observations derived from both the attribution maps and intensity distributions reveal that the observation of the beam made prior to its focus leads to the emergence of larger and more widely distributed inference patterns, such as speckles and fringes. These patterns are likely contributors to the performance gap observed between the models.

Whilst the results presented here offer an important milestone towards neural network control of fibre laser CBC systems, it is important to realise that the SLM used in this work was able to generate stable phase values for the entire length of data collection, and hence did not include phase drifts or other perturbations that one might observe when using fibre lasers.

## Real time phase correction

**Figure 4:**
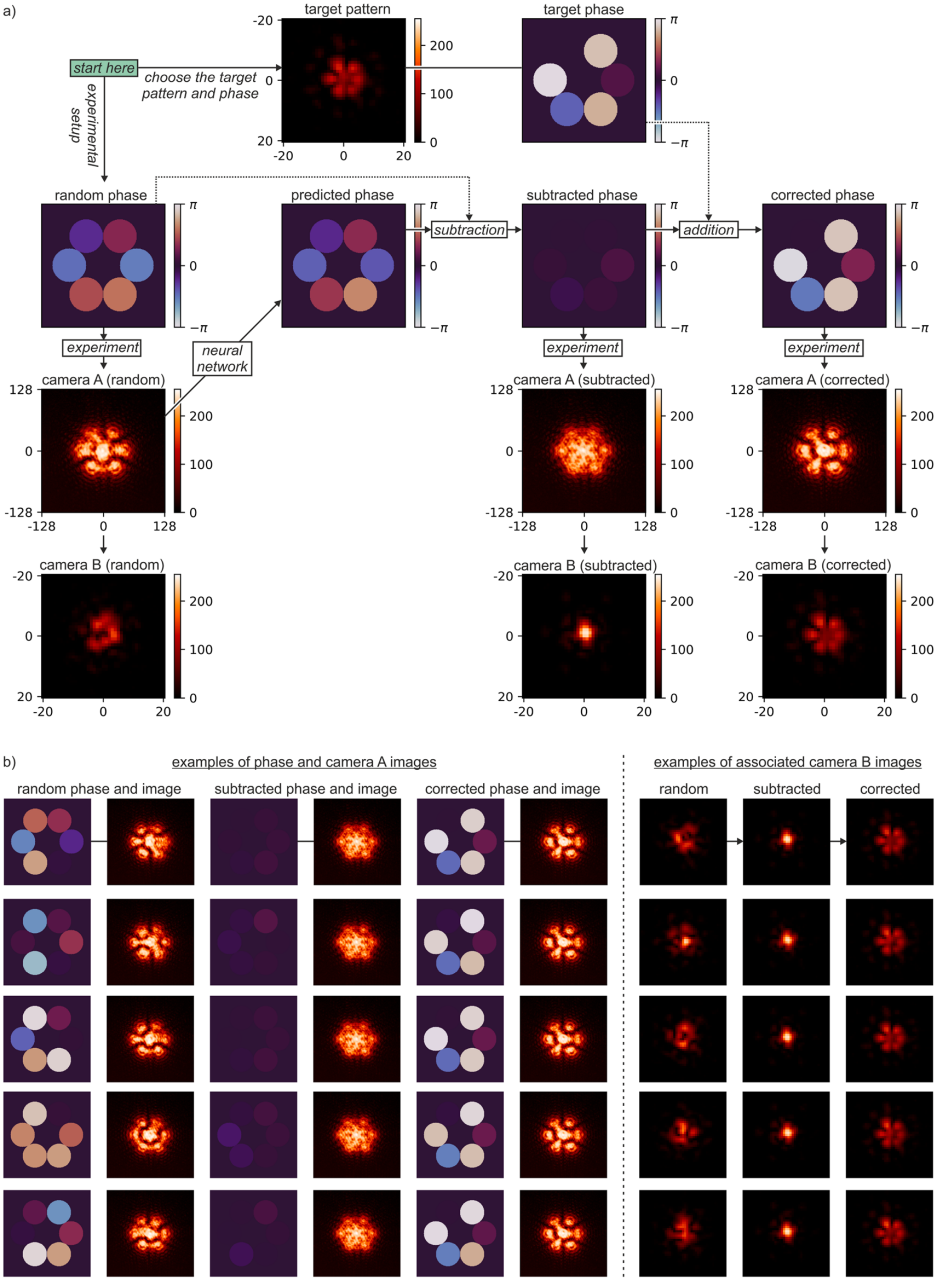
(**a**) Flowchart of the application of deep learning for real-time bespoke beam shaping, and (**b**) examples of random phase values and the progression to the final "petal" beam shape.

After the neural networks were trained, they were applied in real-time on the experimental setup. The results here correspond to real-time experiments enacted up to 17 days after the networks were trained, highlighting the potential for robust and reliable application of this technique in an industrial environment. The primary objective of this work is to experimentally verify our previous theoretical solution for enabling real-time beam shaping^37^ with neural networks. A flowchart of the concept is presented in Fig. 4a, it shows the application of deep learning, first to identify phase values from camera images, and then using those predictions to produce a bespoke "petal" beam shape. At the initial step, labelled “random phase” in Fig. 4a, random phase offsets are assigned to each simulated fibre, generated on the SLM, and the camera A image is recorded. This image is used as an input to the associated neural network, which then predicts the phase values (the "predicted phase"). The camera B image is also recorded and displayed on the figure. The predicted phase values are then subtracted from the random phase values (the "subtracted phase"), and this new set of phases are displayed on the SLM, resulting in updated camera A and camera B images. In the case of a perfect phase prediction, this set of phases would all be zero, hence corresponding to a single interference peak at the focal plane observed on camera B image. The phase profile corresponding to the petal beam (the "target phase") is then added to the current phase (the "subtracted phase") to create the final phase profile (the "corrected phase"). The corrected camera A and camera B images are also shown, where the camera B image clearly shows the desired petal intensity profile. In practice, and as presented later this manuscript, the phase subtraction and phase addition steps can be completed at the same time. Figure 4b shows five examples of random starting phase values and the associated progression to the final petal intensity profile.

## Real time beam shaping

**Figure 5:**
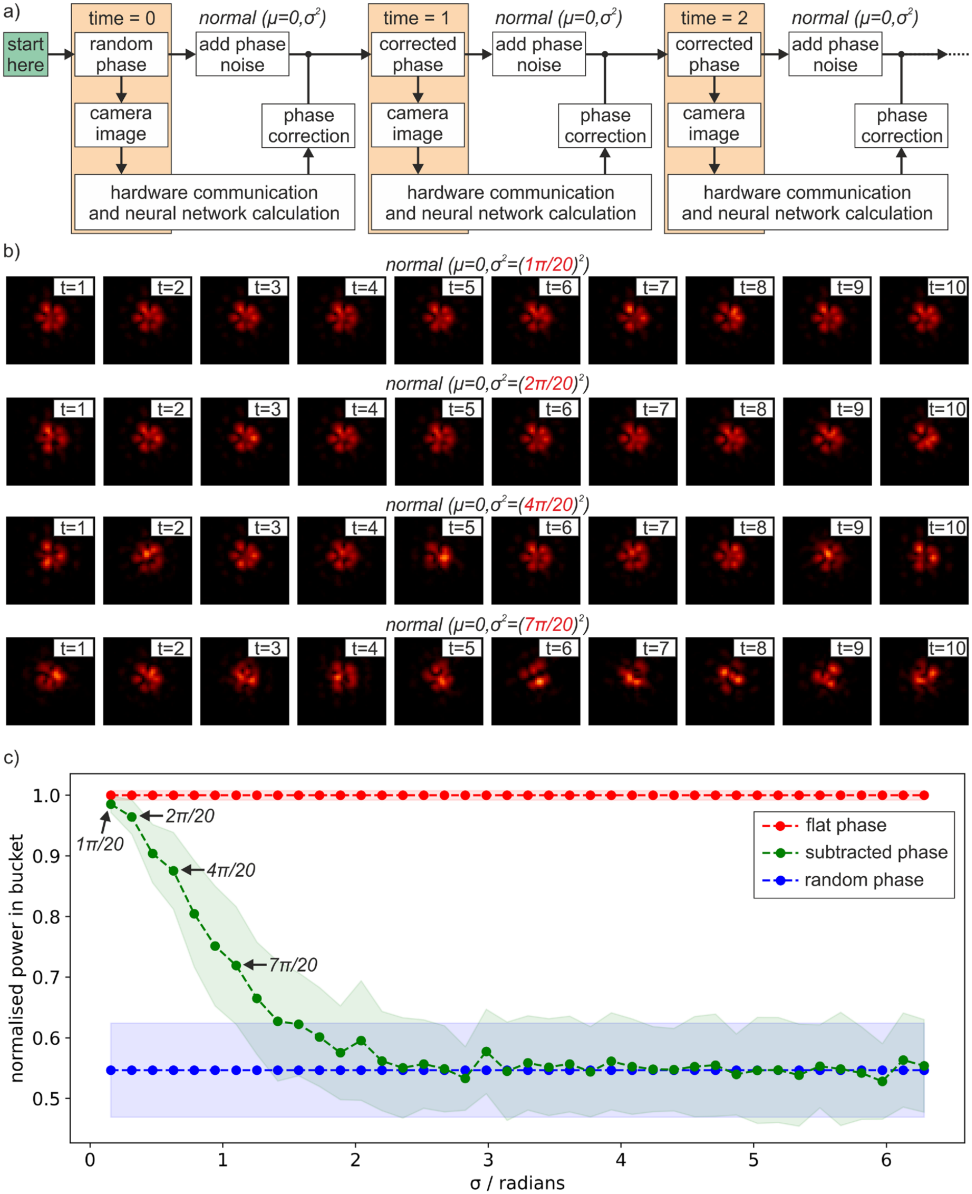
(**a**) Real time concept flowchart for bespoke beam shaping where additional random phase noise is added during the computation and correction step, (**b**) examples of real-time generation of the petal intensity profile under different magnitudes of additional random phase noise, and (**c**) power in the bucket comparison for different standard deviation values.

In general, fibre lasers may have a continuously changing phase value during operation. An important consideration is therefore the degree of additional phase noise that occurs from the time when the camera A image is recorded until the time when the neural network phase corrections are applied. In Fig. 4, for simplicity, there was no additional phase noise applied to the circular regions on the SLM during this time. Figure 5a, however, presents a flowchart that describes the application of deep learning for real-time bespoke beam shaping where additional random phase noise is added to the SLM during the computation and correction time step. In this case, the amount of additional phase noise for each simulated fibre is defined as a normal distribution with an associated mean and standard deviation (denoted as σ in Fig. 5). Whilst in practice there is likely to be a non-zero mean (corresponding to a phase drift process), in this experimental demonstration, a mean of zero was chosen to make comparison of different standard deviation values easier to quantify. The flowchart shows that, for each time step, phase noise is added before the neural network phase correction is applied. Therefore, the likeness of the final petal intensity profile is strongly associated with the standard deviation of the added phase noise, where higher standard deviation values will result in a final intensity profile that may look very different to the desired petal shape. Figure 5b shows experimental data for camera B, for ten consecutive time steps, for standard deviation values of 1π/20, 2π/20, 4π/20 and 7π/20 (two further examples of target intensities for varying degrees of phase noise can be found in the supplementary material Fig. S2). As expected, the lower standard deviation values correspond to strong likeness with the desired petal beam shape. However, above a threshold of approximately 4π/20, the quality of the final petal beam shape is strongly diminished.

Figure 5c shows a power in the bucket measurement for camera B, when the desired beam shape is a single interference peak (i.e., when the phase is "subtracted" but not "corrected" to the petal intensity profile), for different standard deviations of additional phase noise. To provide a metric for the power in bucket measurement, the graph shows the case for a perfect "flat phase" (red line) normalised to a value of one, and the case for "random phase" (blue line), which appears at 0.55. The "subtracted phase" (green line) descends from the flat phase line to the random phase line as the standard deviation is increased. The standard deviation values of 1π/20, 2π/20, 4π/20 and 7π/20 are highlighted on the graph for comparison with b).

Figure 5 therefore underlines that an important consideration in the application of this technique is that the neural network prediction and correction process must be both accurate and fast. It is therefore likely that a primary challenge for this field will now be associated with decreasing the time taken for the neural network inference and correction step. In this work, we deliberately used a neural network that was optimised for lower computational capability. However, further optimisation is almost certainly possible through pruning of the network weights (to make the architecture smaller) and through the application of dedicated hardware. The communication speed for transfer of the camera data to the computer may also be a bottleneck in this process, and so techniques such as hardware image cropping (where the camera only sends data corresponding to a small region on the sensor) and faster hardware interface cards should also be considered. Similarly, the communication speed, the action time for the phase actuators, and the phase noise characteristics of the combined fibre amplifiers will likely also be critical when applying this to a real fibre laser system.

**Figure 6:**
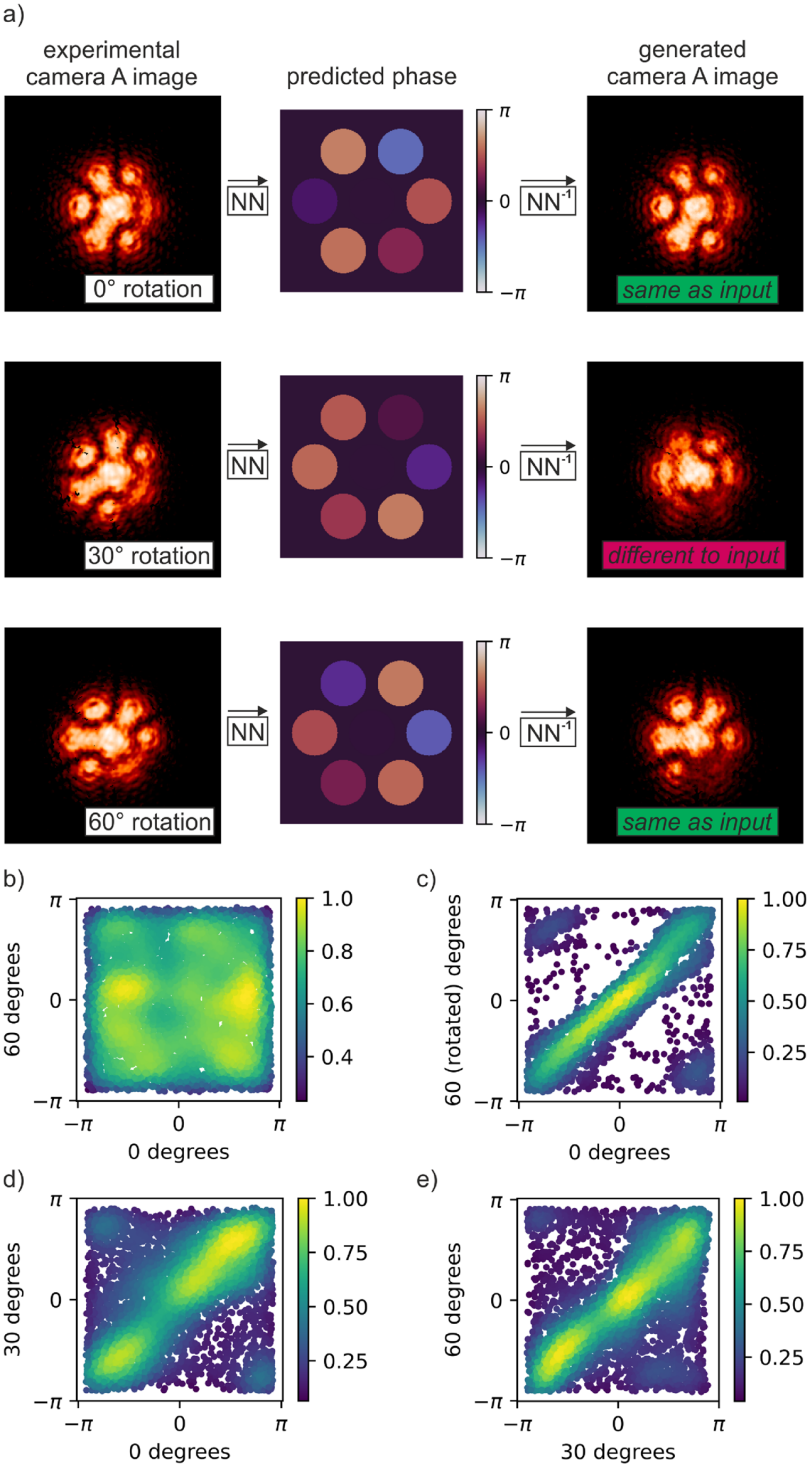
(**a**) Experimental demonstration of the application of two neural networks for determining whether a given intensity pattern is physically possible, showing cases for an experimental camera image that is rotated by 0°, 30° and 60°, and (**b–e**) statistical analysis of predicted phase values when this technique is applied to 900 test examples (see text for explanation).

Figure 6 presents an experimental demonstration of the capability of deep learning for determining whether a desired intensity profile is possible on a given CBC setup. Two additional neural networks were trained, firstly to transform any given camera A image into a phase profile, and secondly to transform any given phase profile into a generated camera A image. The second network can therefore be considered as the "inverse" of the first, and hence they are labelled as ‘NN’ and ‘NN^**-1**^’ on the figure. The two networks were conditional generative adversarial networks that used the "pix2pix" architecture^44^, with input and output image size changed to 256 × 256 pixels. The objective here is to use a "cyclic test", where a camera image is transformed into a phase image before being transformed back into a camera image, to identify whether the input intensity pattern is physically possible on the experimental setup. This technique is possible because (under the assumption of uniform intensity for each fibre) all phase values correspond to a unique intensity pattern, but not all intensity patterns correspond to a unique phase value. (For example, a binary square intensity pattern is not possible with a hexagonal array of seven fibres). Therefore, if an input intensity pattern can be transformed into a phase image (using NN) and then back into the original intensity pattern (using NN^−1^) then the input intensity pattern is physically possible on the experimental setup. If this cyclic test is not successful, then the input intensity pattern is unlikely to be physically possible. Such a technique offers an elegant alternative to the brute-force calculation that would otherwise be needed to determine whether a desired intensity pattern is possible, and hence this technique offers an example of the potential for deep learning in solving complex inverse problems associated with CBC.

Figure 6a presents a camera A image, and the associated predicted phase profile and subsequent generated camera A image, for cases where the camera A image had a 0°, 30° and 60° rotation. The case with 0° rotation corresponds to an experimentally recorded camera image, with a minor transformation to adjust for a skew in the image due to the angle of the SLM. As shown in the figure, the input and generated images for 0° rotation are extremely similar. This is likewise the case for 60°. However, the generated camera A image for 30° is starkly different to the input for 30°. This can be understood because of the six-fold symmetry in the hexagonal close packed array arrangement. When an experimental camera A pattern that is rotated by 30° this breaks the six-fold symmetry and results in an intensity pattern that is not physically possible, whilst an experimental camera A pattern that is rotated by 60° *is* physically possible. Interestingly, the predicted phases for the 60° rotation case are indeed also rotated by 60°, as compared to the 0° case, but this is not the case for the 30° example.

Figure 6b–e show further analysis of the rotation of the phase values, where the predicted phases corresponding to camera A images with 0°, 30° and 60° are compared, alongside the phase profile corresponding to 60° if the array of phase values had been rotated back by 60°. This analysis corresponds to all 900 test camera A cases, with the phase of the central fibre removed from the data. Figure 6b shows that there is almost no correlation between the phase values of 0° and 60° (as expected because they are selected at random). But c) shows that there is strong correlation between the phase values for 0° and the case where the 60° phases have been rotated back. Part c therefore confirms that, in general, the predicted phases rotate by 60° when the input camera A image is rotated by 60°. However, as shown in (d) and (e), the phase predictions at 30 degrees are almost equally correlated with the cases for 0 and for 60 degrees. This indicates that the phase predictions for the 30° case are roughly mid-way between the phase profiles for 0° and 60° rotations. However, whilst the generated camera A image corresponding to the 30° rotation input is possible on the experimental setup (as it corresponds to a set of phase values), this generated camera A image is very different to the associated input camera A image, and hence the cyclic test fails and confirms that this intensity pattern is not physically possible. The small differences between the input and output camera images for 0° and 60° are attributed to the 6° angle of incidence of the HeNe beam onto the SLM (see Fig. 1), which resulted in skew in the experimental camera images that could not be completely corrected.

## Discussion

### Scalability of proposed method

In this work, a set of seven simulated fibres was presented. An important question is therefore the ability for this technique to be scaled up to higher numbers of fibres. Fundamentally, a neural network is a universal function approximator^45^ and hence with sufficient computing power (and associated training data) it should be able to approximate any Borel measurable function, regardless of complexity. Given the rapid increase in GPU capabilities, it seems unlikely that the neural network training and inference will become a limiting factor when scaling up to larger numbers of fibres. Indeed, if the camera image size remains constant, then the input to the neural network will be the same and hence the processing time of the network will be unchanged as more fibres are introduced. It can therefore likely be assumed that the ability to scale up to higher numbers of fibres will not be limited by neural network capability or computing power. Of course, there will be limits to this argument. As the number of fibres increases, the interference fringes between the outer ring of fibres and the central fibre will become smaller, and hence at some point these fringes will be smaller than the pixel resolution of the camera. However, this limit could be alleviated to some extent by using smaller camera pixels, or through additional imaging systems, or even multiple cameras.

However, the interference patterns created by multiple beams scale in complexity as additional sources are added, as each light source will interfere with all other sources. For N light sources, there will be ~ N^2^ interference terms, and hence it could be assumed that the amount of training data required would similarly scale as ~ N^2^. Surprisingly, the results to date do not agree with this hypothesis. In this work 18,114 training data examples was sufficient for 7 fibres, and in previous work^37^ 30,000 examples were sufficient for 19 fibres. Although were unable to test larger numbers of fibres on the experimental setup presented here due to the resolution of the SLM, we have conducted simulations for higher numbers of fibres, which indicate that 50,000 examples are sufficient for 37 fibres, and 50,000 examples for 61 fibres. Whilst this is not an extensive study, the amount of training data required does not appear to scale with the numbers of fibres (particularly as ~ N^2^).

This can be understood via three key results from recent work where a neural network that was tasked with predicting the phase values from CBC intensity patterns was evaluated whilst the training data was kept constant but where the testing data was deliberately modified^38^. Firstly, the results indicated that the neural network could still identify the interference fringes (and hence the phase value) when the amplitudes of the fibres were varying, hence indicating that the network is looking for the shape and not the absolute intensity of the interference fringes. Secondly, the network was unable to predict the phase values when the fibres were moved, hence implying that the network is learning to identify the position of interference patterns. Finally, the network could not predict phase values when the central fibre was removed, implying that the network does not consider the interference between outer fibres to be useful information.

The conclusion is therefore that the network is learning the appearance of fringes that are a consequence of the interference between each outer ring fibre and the central fibre, and learning how to extract these individual sets of fringes from the complete interference pattern and hence identify phase values. As such, each interference pattern contains training data for each fibre, and therefore additional training data might not be needed when the number of fibres is increased. If this argument is true, then this provides a critically important result for the application of neural networks for control of CBC systems, as the time needed for collecting training data may not increase as additional fibres are added.

### Resilience of the proposed method to intensity and central wavelength instabilities

In this work, whilst we used a stabilised HeNe (set to intensity-stabilisation mode) to minimise the effect of thermal drift on the output power during data collection, previous results have shown that this is generally not necessary^38^ as the phase prediction accuracy is not degraded as a result of random intensity fluctuations that occur independently on each fibre, even for ~ 75% reductions in intensity (whilst the neural network has been shown capable of identification of phase values when the fibre amplitudes are varying, this will likely only be true for camera images where there is no image saturation). However, in a real fibre laser CBC system, both the power and central wavelengths are liable to drift during operation. Whilst power variations are manageable (as they only change the magnitude of fringes), the evidence suggests that shifts in wavelength might cause significant reductions in the accuracy of phase predictions. Changes in wavelength may change the position and/or spatial frequency of fringes for fixed phase values, and previous results^38^ showed that the network cannot make accurate phase predictions when the position of fringes are changed. In addition, the temporal coherence may need to be considered, as temporal beating might be observed on the camera images, depending on integration times and wavelength shifts. Whilst additional training data could be included, it is possible that techniques for minimising wavelength variation will be an important factor in the practical implementation of such a neural-network-controlled system.

## Conclusions

This work presents an experimental demonstration of single-step bespoke beam shaping on a coherent beam combination system, using seven beams created by a spatial light modulator. The phase correction method was achieved by using deep learning to identify the phase of each beam directly from a camera image of the combined intensity pattern. This process was operated in a closed-loop feedback process and used to demonstrate the creation of a consistent "petal" beam shape despite the presence of random phase noise.

### Supplementary Information

Supplementary Figures.

## Data Availability

Data underlying the results presented in this paper are available in https://doi.org/10.5258/SOTON/D2974.
