# Supplementary material for: Single-step phase identification and phase locking for coherent beam combination using deep learning
Source: Sci Rep. 2024 Mar 29;14:7501. doi: 10.1038/s41598-024-58251-z (PMC10980735; doi:10.1038/s41598-024-58251-z)
Supplement: Supplementary file 1 — Supplementary Figures. [file 41598_2024_58251_MOESM1_ESM.docx]

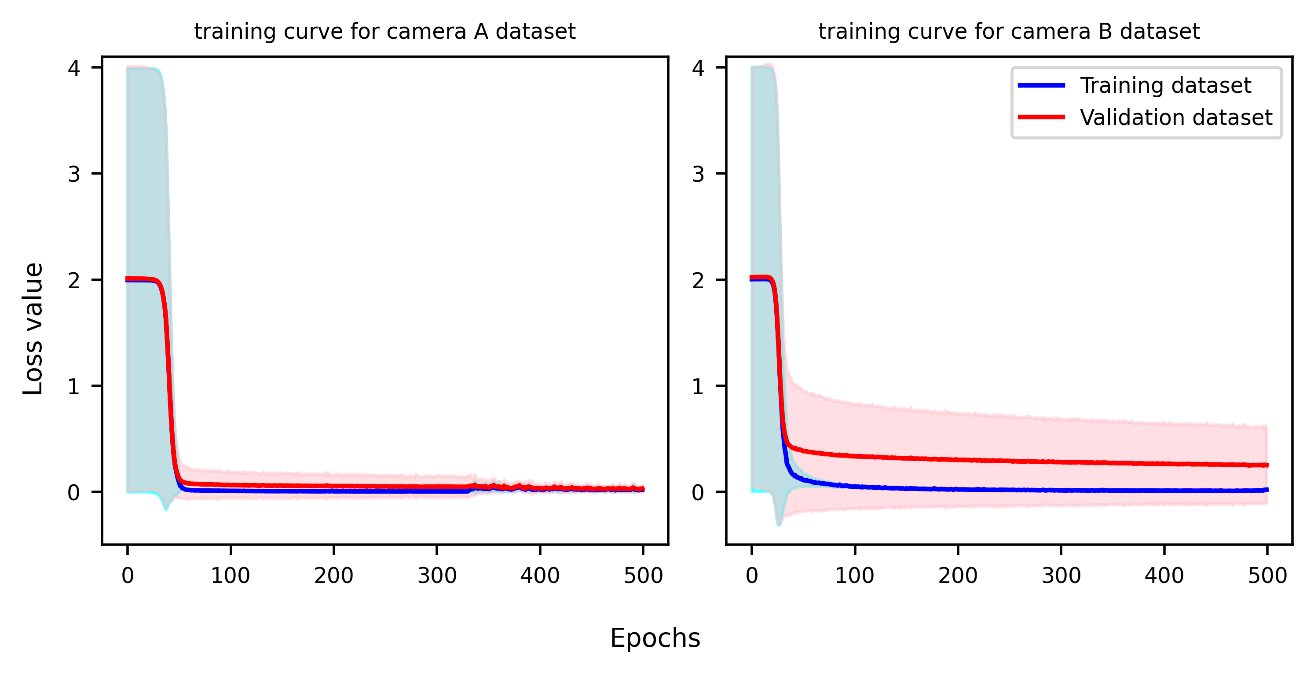


Figure S1. Training curves for the camera A dataset (left) and camera B dataset (right).


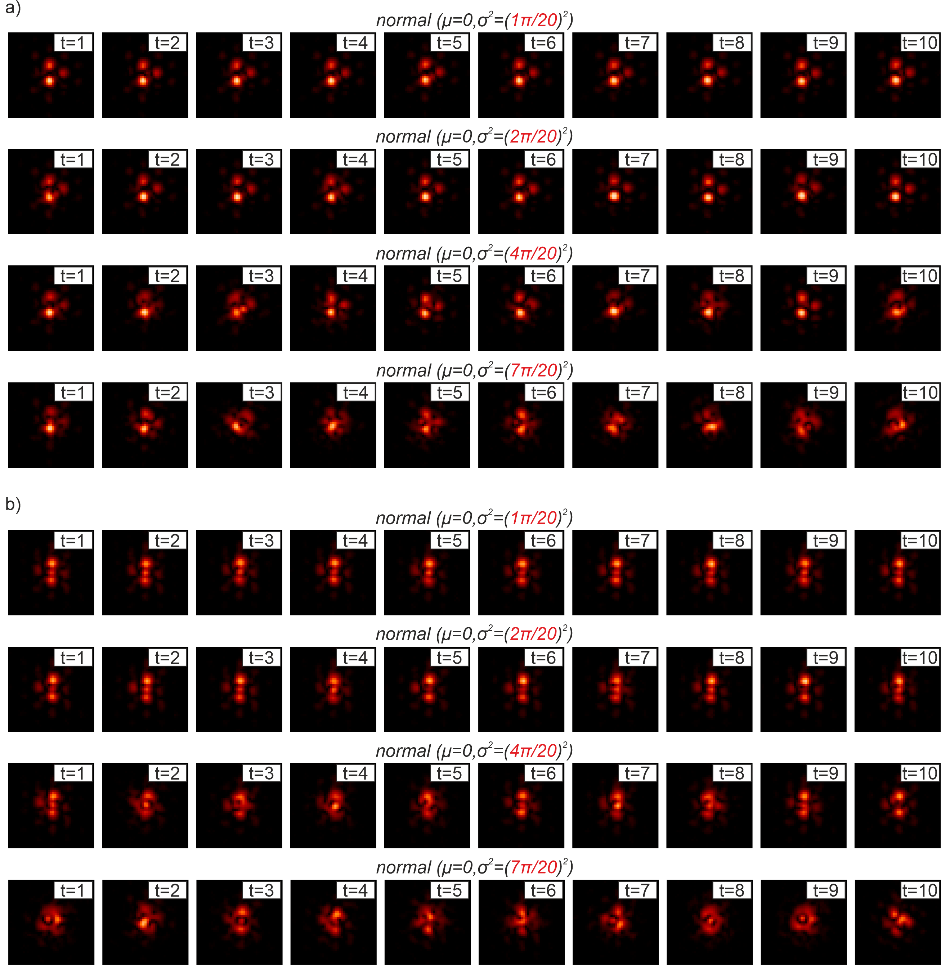


Figure S2. Experimental target intensity patterns for a) clover and b) sandwich designs, under varying degrees of phase noise, using the single-step correction process explained in figure 5.
